# The impact of the COVID-19 pandemic on individuals with mental illness: A two-wave survey of 1180 patients

**DOI:** 10.1192/j.eurpsy.2021.270

**Published:** 2021-08-13

**Authors:** Y. Gil, M. Speed, O. Jefsen, P. Kølbæk, S. Østergaard

**Affiliations:** 1 Department Of Affective Disorders, Aarhus University Hospital - Psychiatry, Aarhus N, Denmark; 2 Institut For Klinisk Medicin - Transitional Neuropsychiatry, Aarhus University, Aarhus C, Denmark; 3 Psychosis Research Unit, Aarhus University Hospital - Psychiatry, Aarhus N, Denmark; 4 Department Of Clinical Medicine, Aarhus University, Aarhus, Denmark

**Keywords:** pandemic, Survey, Mental illness, Benchmark

## Abstract

**Introduction:**

The crisis caused by the ongoing COVID-19 pandemic is affecting the lives of billions of people across the world. Individuals with mental illness are suspected to be particularly affected by the societal consequences of the pandemic, but there is very limited data on this important aspect.

**Objectives:**

The aim of this study is to gauge the longitudinal impact of the COVID-19 pandemic on the psychological well-being and symptom levels of individuals receiving treatment for mental disorder in psychiatric hospital services.

**Methods:**

We are in the process of conducting a two-wave, questionnaire-based survey among patients with mental disorders receiving treatment in the psychiatric hospital services of the Central Denmark Region. The first wave was conducted in July 2020 and had 1180 respondents representing all major diagnostic categories. The main finding was that the majority of the respondents reported that their mental health had deteriorated during the COVID-19 pandemic. We are currently planning the second wave of the survey, which will be fielded in the fall of 2020. Here, we will reassess the mental health of the respondents from wave 1.

**Results:**

Will be presented at the meeting.

**Conclusions:**

Longitudinal studies of the impact of the COVID-19 pandemic of mental health are lacking. We therefore expect that the findings of this study will be of significant interest to the field.

**Disclosure:**

No significant relationships.

